# Supplementary material for: Biological and experimental factors that define the effectiveness of microbial inoculation on plant traits: a meta-analysis
Source: ISME Commun. 2024 Oct 14;4(1):ycae122. doi: 10.1093/ismeco/ycae122 (PMC11538580; doi:10.1093/ismeco/ycae122)
Supplement: Supplementary_information_ycae122 [file supplementary_information_ycae122.docx]

**Biological and experimental factors that define the effectiveness of microbial inoculation on plant traits: a meta-analysis**

Hamed Azarbad***** and Robert R. Junker

Evolutionary Ecology of Plants, Department of Biology, University of Marburg, Karl-von-Frisch-Strasse 8, 35043, Marburg, Germany.


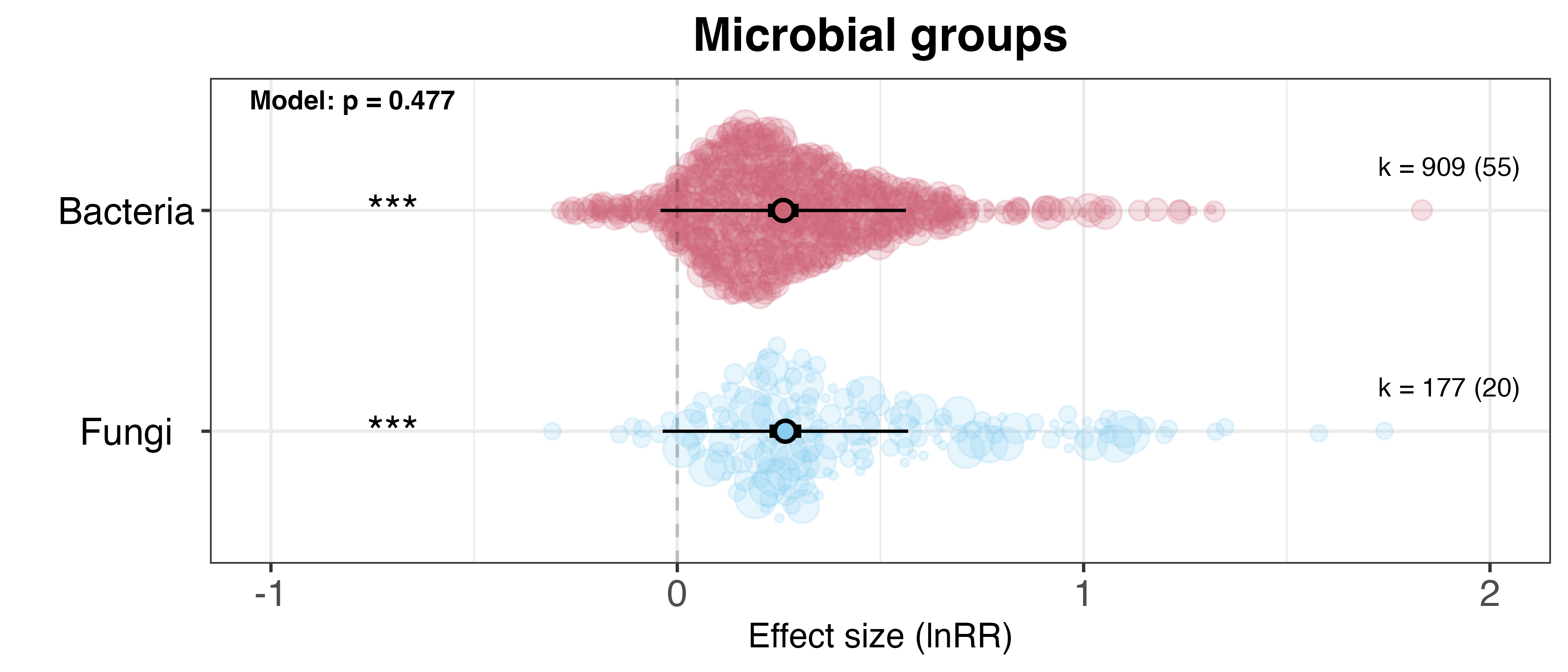


**Fig S1**. Effect of microbial groups (bacteria and fungi) on the strength of the effect size.


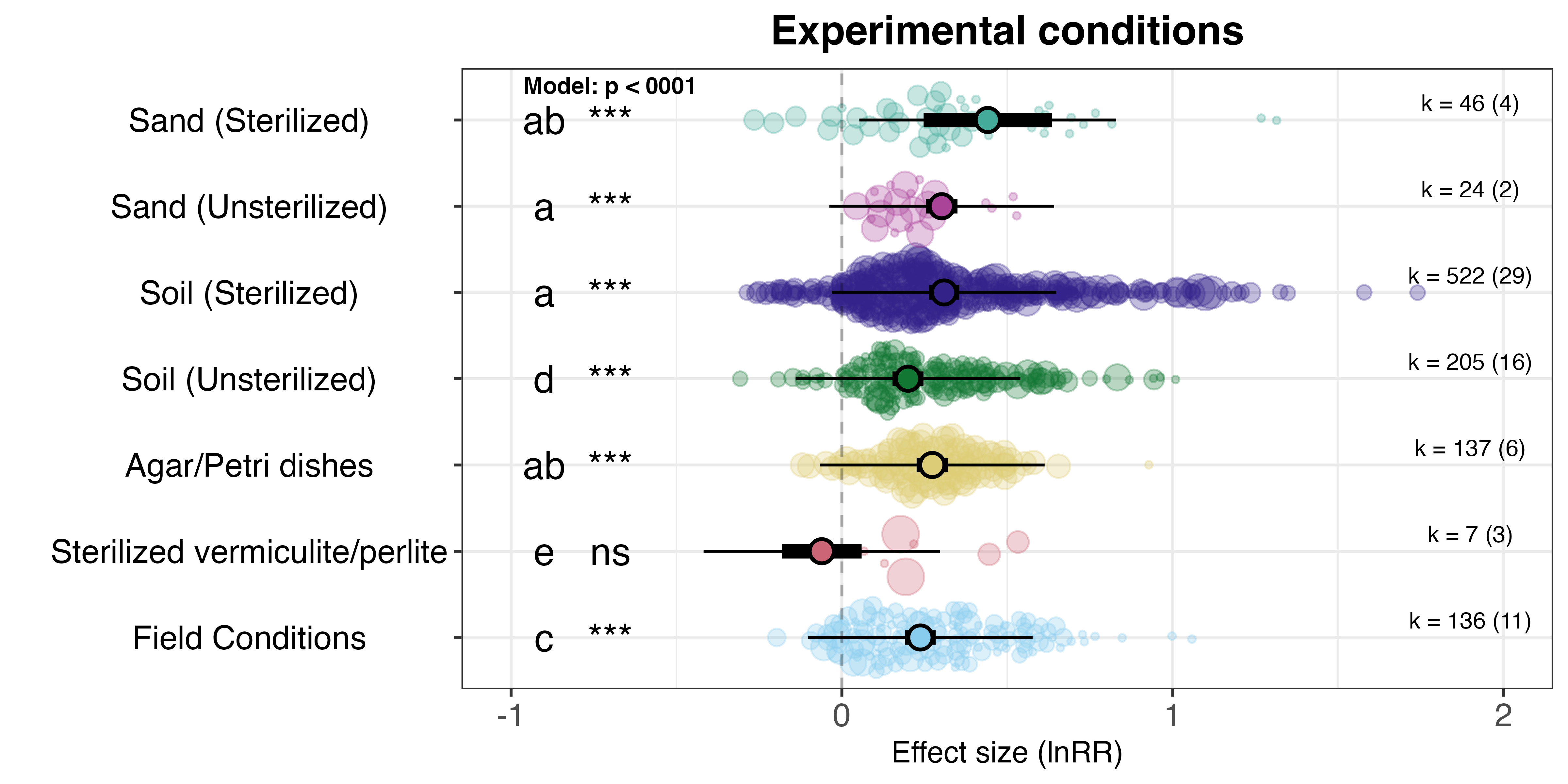


**Fig S2**. Effect of experimental conditions on the strength of the effect size.
